# Safety of Adeno-Associated Viral Vectors in Gene Therapy: Mechanisms of Toxicity, Clinical Risks, and Strategies for Their Minimization

**DOI:** 10.3390/ijms27114818

**Published:** 2026-05-27

**Authors:** Tatiana S. Tsaregorodtseva, Maria A. Radyukhina, Aisylu I. Ayupova, Valeriya V. Solovyeva, Albert A. Sufianov, Galina Z. Sufianova, Albert A. Rizvanov

**Affiliations:** 1 Institute of Fundamental Medicine and Biology, Kazan Federal University, 420008 Kazan, Russia; tascaregorodceva@kpfu.ru (T.S.T.); maaradyukhina@kpfu.ru (M.A.R.); ayimullagulova@kpfu.ru (A.I.A.); vavsoloveva@kpfu.ru (V.V.S.); 2 Department of Neurosurgery, Sechenov First Moscow State Medical University of the Ministry of Health of the Russian Federation (Sechenov University), 119991 Moscow, Russia; sufianov_a_a@staff.sechenov.ru; 3 The Research and Educational Institute of Neurosurgery, Peoples’ Friendship University of Russia, 117198 Moscow, Russia; 4 Department of Pharmacology, Tyumen State Medical University, 625023 Tyumen, Russia; sufarm@mail.ru; 5 Division of Medical and Biological Sciences, Academy of Sciences of the Republic of Tatarstan, 420111 Kazan, Russia

**Keywords:** adeno-associated virus, gene therapy, vector toxicity, immunogenicity, hepatotoxicity, immunosuppression, capsid engineering, clinical safety

## Abstract

Adeno-associated viral (AAV) vectors have established themselves as a promising platform for genetic material delivery in clinical practice, evidenced by regulatory approval of multiple therapeutics. Despite proven therapeutic efficacy, safety concerns remain a critical limitation requiring systematic analysis. This review analyzes clinical data to identify mechanisms of toxicity, clinical risks, and strategies for their minimization in AAV gene therapy. The study examines dose-dependent toxicity, immune responses, and organ-specific burdens associated with systemic and local administration routes. Analysis reveals a clear correlation between systemic delivery efficacy and dose-dependent toxicity, with principal mechanisms including capsid-directed immune responses, hepatic burden, and complement system activation leading to thrombotic microangiopathy. Key determinants of the safety profile include pre-existing neutralizing antibodies, vector dose, serotype selection, and patient baseline conditions. Contemporary strategies for toxicity minimization are evolving from reactive management toward proactive risk mitigation, including prophylactic immunosuppressive regimens, vector engineering to alter tropism or reduce immunogenicity, and rigorous post-infusion monitoring. Integration of improved vector constructs, rational immunosuppressive regimens, and rigorous post-infusion surveillance has the potential to expand the therapeutic window of AAV-based gene therapy, achieving an optimal balance between efficacy and safety for a broader patient population.

## 1. Introduction

Recombinant adeno-associated viral vectors (rAAV) have established themselves as a leading platform for in vivo gene delivery due to their high tissue tropism, low pathogenicity, and capacity for sustained transgene expression. The successful regulatory approval of several therapeutic products (e.g., Zolgensma, Luxturna, Hemgenix, and Elevidys) has confirmed the high clinical efficacy of this technology [1,2,3]. However, the widespread clinical application of AAV-based vectors is associated with significant safety challenges. The administration of high viral doses, often required to overcome biological barriers and achieve transduction across extensive tissue areas, frequently leads to dose-dependent toxic effects, including hepatotoxicity, complement system activation, thrombotic microangiopathy, and robust immune responses against the capsid or transgene product [1,4]. Although several reviews have addressed the general biological properties of adeno-associated viruses, a systematic analysis of specific toxicity mechanisms and clinical risk management strategies remains highly relevant, particularly in light of recent clinical data. The aim of this review is to provide a comprehensive analysis of the factors determining AAV safety, elucidate the mechanisms underlying immune-mediated and organ-specific adverse effects, and evaluate current mitigation strategies—ranging from rational immunosuppression regimens to engineered capsid modifications—to expand the therapeutic window of gene therapy.

### 1.1. AAV-Based Gene Therapy: Key Milestones in Scientific and Clinical Development

The starting point for the use of adeno-associated viruses (AAV) in gene therapy was their discovery in the 1960s as an undesirable contaminant of laboratory adenovirus cultures grown in kidney tissue cells of rhesus macaques [5]. Its unique biological properties—obligate dependence on co-infection with a helper virus (such as adenovirus, herpes simplex virus, cytomegalovirus (CMV), or papillomavirus) for replication and lack of pathogenicity in host cells—from the very beginning defined its special status among viral agents [6,7]. A fundamental milestone that laid the foundation for converting AAV into a delivery vector was the determination of the nucleotide sequence of the inverted terminal repeats (ITRs) of AAV2 DNA, which enabled the concept of rAAV, in which all viral genes (rep and cap) are replaced by a therapeutic genetic cassette retaining only the ITRs necessary for replication and packaging [5,8].

The first clinical trials of rAAV, conducted in the 1990s for the treatment of cystic fibrosis, confirmed the basic safety of the platform but also revealed its initial limitations: insufficient efficacy due to low transduction, immune response, and a limit on the size of packaged DNA (~4.7 thousand base pairs (kbp)) [8,9,10]. From the initial identification of three serologically distinct serotypes in 1966, scientific progress led to a qualitative leap in the 2000s, when the discovery and characterization of numerous natural isolates (AAV1-AAV9, AAVrh10, etc.) with unique tropism for key target tissues (liver, central nervous system (CNS), skeletal muscles, etc.) laid the foundation for targeted gene therapy [7,11].

Achieving stable and long-term transgene expression after a single administration became possible due to the simultaneous solution of three fundamental tasks. Firstly, scalable production methods were developed that completely exclude the use of infectious helper virus, which allowed obtaining high titers of purified recombinant AAV particles suitable for clinical application. Secondly, overcoming the limiting step of second-strand DNA synthesis through the creation of self-complementary vectors (scAAV) significantly increased the efficiency and speed of transduction. Thirdly, the construction of tissue-specific promoters ensured targeted expression of the therapeutic gene strictly in target cells, minimizing systemic effects [12].

The result of this long-term research was the emergence of the first clinical candidates, including Glybera—the world’s first rAAV1-based gene therapy drug, approved in 2012 for the treatment of a rare inherited lipid metabolism disorder [8]. Despite subsequent commercial difficulties, this event marked the formal recognition of the maturity and potential of the AAV platform [13].

### 1.2. The Role of AAV Vectors in Modern Gene Medicine

The process of AAV penetration into the cell begins with its binding to glycosylated receptors on the cell surface [14]. After that, the virus enters the cell through clathrin-mediated endocytosis [15]. It then moves through the cytosol using elements of the cytoskeleton [16]. In the acidic environment of the endosome, the capsid proteins VP1 and VP2 change their structure, which helps the virus escape from the endosome [17]. The AAV is then directed to the nucleus, where its genetic material is released. However, a portion of viral particles may be degraded in proteasomes, which reduces the overall efficiency of transduction [18]. In contrast to adenoviruses or retroviruses, rAAV can be characterized as predominantly non-integrating or episomal, with low-frequency random integration, which minimizes the risk of insertional mutagenesis and ensures a predictable safety profile [19,20].

A key advantage of AAV vectors is the possibility of tissue-specific transduction, determined by the natural diversity of capsids. As of 2025, the diversity of known AAVs has expanded significantly. In nature, more than 100 natural serotypes and isolates of AAV have been identified, including representatives from both human and non-human primates, as well as other mammals [3,21].

One of the central challenges in the application of AAV remains its limited cargo capacity, which hinders the delivery of large genes, such as dystrophin in Duchenne muscular dystrophy (DMD). The length of mature dystrophin mRNA is ~14 kbp, which accounts for less than 1% of the gene size [22]. To overcome this limitation, strategies using shortened dystrophins—truncated but functionally active versions of the gene that fit within the vector capsid—have been developed. In a clinical trial based on AAVrh74, transduction of skeletal muscles and myocardium in patients with DMD led to sustained expression of microdystrophin and improved motor function (NCT03769116) [23].

### 1.3. Modern Safety Challenges

The evolution of AAV application has been accompanied by a deepened understanding of associated risks. The tragic death of 18-year-old Jesse Gelsinger in 1999 from an acute immune reaction to an adenoviral vector became a harsh lesson for the entire field of gene therapy [24]. This incident occurred despite promising preclinical data and led to the development of systemic inflammatory response syndrome and multiorgan failure, resulting in a fatal outcome 98 h after infusion. This case highlighted not only the immunogenicity of viral vectors but also the critical limitations of animal models in predicting individual human responses, as well as the existence of a narrow therapeutic window and a “steep toxicity curve” for administering high doses [25].

In the context of AAV, it became evident that administering high vector doses (≥5 × 10^13^ viral particles/kilogram (vp/kg)), necessary for treating diseases affecting extensive tissues, can be associated with serious, and sometimes fatal, complications, including hepatotoxicity, thrombotic microangiopathy (TMA) caused by complement system activation, cardiotoxicity, and neurotoxicity affecting dorsal root ganglia [26,27]. These challenges have shifted the focus of modern research toward addressing key tasks: targeted capsid engineering to minimize nonspecific delivery and the development of adaptive protocols for concomitant therapy to control immune responses [28].

### 1.4. Clinical Experience with AAV Therapies

The clinical trajectory of AAV therapies in recent years demonstrates an evolution from conceptual proof to a complex medical technology, the success of which is determined not only by the molecular design of the vector but also by the patient’s immunological status, delivery route, dose per kilogram of body weight, and adequacy of concomitant therapy.

The first registered drug was Glybera—an rAAV1 carrying a functional copy of the lipoprotein lipase (LPL) gene under the control of a CMV promoter [29]. Approved by the European Medicines Agency (EMA) in October 2012 for the treatment of homozygous LPL deficiency. The product was never approved by the Food and Drug Administration (USA) (FDA) and was commercially withdrawn in October 2017 due to limited commercial viability and high cost. It required multiple injections with a total dose of up to 3 × 10^12^ vp into the muscles of the lower extremities [30]. Despite a moderate and transient reduction in triglyceride levels in some patients, the therapy did not provide sustained expression of functional LPL in the systemic circulation, as confirmed by the absence of a significant increase in post-heparin LPL activity. Although 4 out of 9 patients showed a reduced frequency of pancreatitis during 1.5 years of follow-up, the effect was inconsistent, highlighting the limited efficacy of local intramuscular delivery for a systemic metabolic disorder [31]. The commercial withdrawal of the drug in 2017 was due not only to its extreme cost ($1 million) but also to the inefficacy of local delivery for this disease, underscoring the need for global vector distribution in multiorgan disorders [13,32].

A different approach was implemented in Luxturna—an rAAV2 carrying the functional retinal pigment epithelium-specific 65 kDa protein (RPE65) gene for delivery to retinal pigment epithelium cells [33]. FDA (2017) and EMA (2018) approval of Luxturna followed the phase III trial (NCT00999609), in which subretinal administration of 1.5 × 10^11^ vp to each eye led to a significant improvement in patients’ ability to navigate under low-light conditions at 1 year. Thirteen out of 20 (65%) patients in the treatment group achieved the ability to orient themselves at minimal light levels, whereas no patients in the control group did. No systemic immune reactions to the capsid were reported [34].

A qualitative leap occurred with the transition to systemic delivery of rAAV. Zolgensma—a drug based on rAAV9 carrying a copy of the survival motor neuron (SMN)1 gene—was approved by the FDA in May 2019 based on the STR1VE-US trial (NCT03306277) and by the EMA in May 2020 based on the STR1VE-EU trial (NCT03461289). In infants with biallelic mutations in SMN1 and copies of SMN2, a single intravenous infusion of 1.1 × 10^14^ vp/kg provided 91% survival. However, seven patients exhibited elevated hepatic aminotransferases, requiring a course of prednisolone [35].

A similar trade-off was observed in the treatment of hemophilia B with Hemgenix—an rAAV5 carrying a factor IX (FIX)-Padua (R338L, hyperactive variant) cassette, approved by the FDA in November 2022 and by the EMA in July 2023 [36,37]. At a dose of 2 × 10^13^ vp/kg, a mean FIX level of 36% was observed at 6 months, along with a 96% reduction in bleeding events. However, elevated alanine aminotransferase was observed in 20% of patients (NCT03587116).

Unlike hemophilia B, hemophilia A (factor VIII (FVIII) deficiency) presents greater therapeutic complexity due to the heightened immunogenicity of the transgenic protein and the necessity of using a truncated form of FVIII with the B-domain deleted, which possesses reduced specific activity. Roctavian is an rAAV5 vector with a single-stranded DNA cassette containing a hepatocyte-specific promoter and a codon-optimized FVIII sequence. The pivotal phase 3 GENEr8-1 trial (NCT03370913) supported its regulatory approval: EMA in August 2022 and FDA in June 2023. The size of the expression cassette exceeds the natural packaging capacity of AAV, leading to genomic instability and increased heterogeneity of vector particles [38]. These features contribute to increased capsid load, which may lead to activation of the alternative complement pathway [27].

The expansion of therapeutic indications has driven the development of drugs for treating neuromuscular pathologies. Elevidys (delandistrogene moxeparvovec), an rAAVrh74-based vector carrying a microdystrophin gene, received FDA accelerated approval in June 2023, which was converted to full approval in June 2024 following confirmatory trial data [1]. Because the full-length dystrophin gene exceeds the vector’s packaging capacity, a functionally active truncated version is employed. Administration requires mandatory pre-screening for antibodies against AAVrh74 (infusion is not recommended at titers ≥ 1:400) and is consistently associated with the development of a humoral immune response in all patients [39]. The clinical safety profile is characterized by dose-dependent hepatotoxicity, transient thrombocytopenia, and potential cardiotoxicity, necessitating intensive monitoring of liver function, troponin I, and platelet counts, alongside a 60-day corticosteroid regimen [40,41].

For the treatment of the rare neurotransmitter disorder aromatic L-amino acid decarboxylase deficiency, the therapies Upstaza (EMA, 2022) and Kebilidi (FDA, 2024) are utilized. Both contain the same active substance—eladocagene exuparvovec—based on rAAV2. In contrast to systemic approaches, the vector is administered via intraputaminal injection, enabling localized transduction of striatal neurons with minimal systemic exposure. Despite the immune-privileged status of the central nervous system, all patients exhibit a sustained increase in binding and neutralizing antibody titers against the AAV2 capsid following administration [42]. The most frequent adverse events include dyskinesias, hypersalivation, fever, and insomnia. Clinical use of Upstaza is restricted in patients with baseline anti-AAV2 antibody titers exceeding 1:50 [43,44].

As of 2026, nine distinct AAV-based gene therapies have received regulatory approval globally. Of these, eight remain commercially available, while Glybera (EMA, 2012) was voluntarily withdrawn from the market in 2017. Jurisdictional approvals differ: Upstaza (EMA, 2022) and Kebilidi (FDA, 2024) are region-specific, whereas Luxturna, Zolgensma, Hemgenix, Roctavian, and Elevidys hold dual FDA/EMA authorizations between 2017 and 2024. The ninth approved product in the AAV gene therapy class is Beqvez (fidanacogene elaparvovec), which received FDA authorization in December 2024 for the treatment of hemophilia B [1]. This vector employs an engineered AAV-Spark100 capsid, optimized for enhanced hepatocyte transduction and delivery of a hyperactive factor IX variant. As with other systemic AAV therapies for coagulopathies, Beqvez’s safety profile is marked by dose-dependent transaminase elevations in a subset of patients, mandating the use of immunosuppression protocols and prolonged monitoring of hepatobiliary function post-infusion [43].

Thus, accumulated clinical experience has revealed a general pattern: the efficacy of systemic AAV therapies directly correlates with dose, but is simultaneously associated with the risk of dose-dependent toxicity mediated by immune responses to the capsid and hepatic burden.

## 2. AAV Serotypes and Tropism

**Figure 1:**
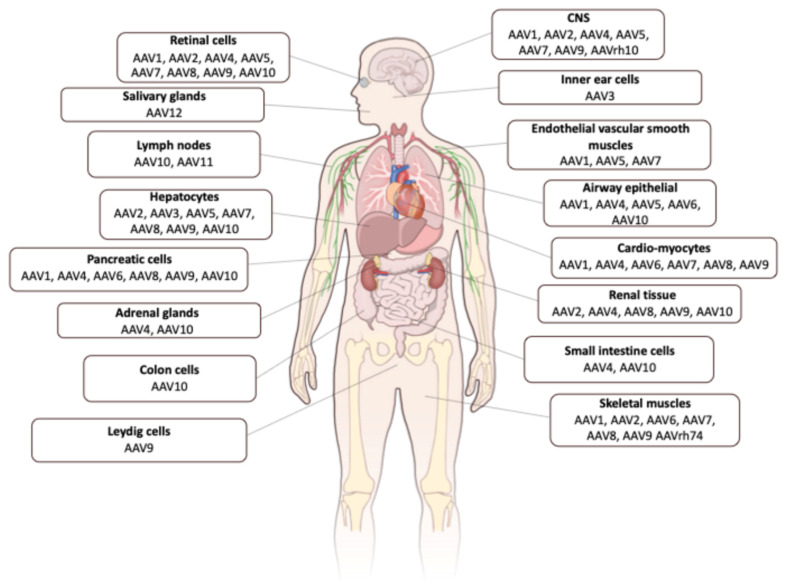
Map of AAV serotype tropism to human organs and tissues. Illustration from NIAID NIH BioArt Source (https://bioart.niaid.nih.gov/bioart/519, https://bioart.niaid.nih.gov/bioart/228, https://bioart.niaid.nih.gov/bioart/229, all accessed on 24 May 2026).

Natural AAV serotypes exhibit pronounced differences in tissue distribution, determined by structural features of the capsid proteins VP1, VP2, and VP3, which govern interactions with cellular receptors and co-receptors [3,45]. Below is a characterization of key natural isolates most frequently used in clinical and preclinical studies (Figure 1).

AAV1 was one of the first AAV vectors used in clinical practice [6]. It binds to N-linked sialic acid and exhibits high tropism for skeletal muscles, CNS, respiratory tract, retina, and pancreas. It is used in experimental therapies for muscular dystrophy and heart failure [1]. AAV2 was the first cloned serotype. It uses heparan sulfate proteoglycans (HSPG) as the primary receptor and fibroblast growth factor receptor 1 (FGFR1), hepatocyte growth factor receptor (HGFR), laminin receptor (LamR), CD9, and tetraspanin as co-receptors. It efficiently transduces retinal cells, CNS, kidney, and liver cells but exhibits low resistance to human neutralizing antibodies (NAb) [46,47]. It was widely used in early clinical trials [1,48]. AAV3 is similar to AAV2 but demonstrates enhanced transduction of hepatocytes and inner hair cells of the cochlea in vivo in mice [11], particularly in the presence of the S663V + T492V mutation in VP1 [49]. It binds to HSPG and is rarely used in clinical settings due to high seroprevalence of antibodies [3,50].

AAV4 uses α2-3O-linked sialic acid as its primary receptor and exhibits narrow tropism for endothelial cells in the stomach, urinary bladder, adrenal glands, pancreas, brain, small intestine, and thymus, while practically not transducing the liver [51]. This specificity makes it a candidate for ventricular delivery [52]. AAV5 exhibits the greatest structural divergence among serotypes and uses N-linked sialic acid and platelet-derived growth factor receptor as functional receptors [1]. It demonstrates moderate transduction efficiency, particularly in oligodendrocytes, and is preferable for intraparenchymal delivery due to its limited ability to cross the blood–brain barrier (BBB) compared to AAV9 [53,54].

AAV6 is a natural homolog of AAV1, with high homology of capsid proteins, and uses N-linked sialic acid and HSPG for binding. It demonstrates high transduction efficiency in skeletal muscles and cardiomyocytes, surpassing AAV1 and AAV9 in some models. It is also effective for delivery to the nasal and bronchial epithelium [3,55]. Furthermore, AAV6 demonstrates high transduction efficiency in adipose tissue and preadipocytes, outperforming other serotypes such as AAV5, AAV8, and AAV9, indicating broad tissue tropism [56]. AAV7, like AAV8, demonstrates similar but less pronounced tropism for hepatocytes [57]. Its liver transduction efficiency is significantly lower than that of AAV8, and it is rarely used in modern preclinical or clinical programs, having been superseded by more efficient serotypes [58,59]. AAV8 binds via the LamR co-receptor and exhibits the highest hepatotropism among all serotypes [1]. It is resistant to NAbs [60] and is used in therapies for inherited liver diseases, including hemophilia [61]. It possesses moderate ability to transduce skeletal muscles and pancreatic endocrine cells [62,63].

AAV9 uses galactose as its primary receptor and possesses a unique ability to cross the BBB in newborns and infants. Efforts to enhance AAV9’s ability to cross the BBB have led to the creation of modified variants such as AAV.CPP.16 and AAV.CPP.21, which demonstrates increased CNS transduction efficiency in rodents and primates through enhanced transcytosis across the BBB [64]. AAV9 transduces a wide spectrum of cells: neurons, astrocytes, hepatocytes, cardiomyocytes, and endothelium. This serotype formed the basis of Zolgensma [65]. AAVrh10, identified in tissues of non-human primates, demonstrates broad and sustained neurotropism upon intrathecal administration in adults. It efficiently transduces neurons of the spinal cord, cerebellum, cortex, and basal ganglia, surpassing AAV9 in uniformity of distribution in the CNS after local delivery [53,66]. Furthermore, it efficiently transduces the subepithelial nerve plexus and can be transported to neuronal ganglia, as shown in corneal nerve regeneration models, indicating its broad potential for neuronal targeting [67,68].

AAVrh74 is an isolate obtained from non-human primates, characterized by high affinity for skeletal muscles with relatively low hepatotropism [41]. This allows for the hepatic burden in muscle-targeted therapy to be reduced. It is used in clinical programs for DMD [23].

## 3. Routes of Administration

The route of administration of rAAV-based vectors is a critical factor determining their biodistribution, systemic exposure, and immunological visibility, which directly influences both therapeutic efficacy and the risk of complications. Administration methods have their own specific features in terms of tissue exposure, required dosing, immune activation, and potential toxicity (Table 1). Systemic administration ensures broad distribution but is associated with high hepatic uptake, dose-dependent toxicity, and strong immune responses, whereas intrathecal and local administration provide more targeted delivery with reduced systemic exposure and immunogenicity, although it is also associated with certain risks and limitations [69,70,71,72,73,74]. Understanding these differences is essential for optimizing gene therapy strategies and minimizing adverse effects.

### 3.1. Systemic (Intravenous and Intra-Arterial) Administration

Systemic administration of rAAV leads to broad distribution of the vector, with the liver being the primary site of uptake due to its high affinity for most AAV serotypes. Achieving therapeutic levels in non-hepatic tissues often requires high doses, resulting in disproportionate accumulation in hepatocytes and an increased risk of hepatotoxicity, systemic immune activation (including CD8^+^ T-cell and complement responses), and adverse effects such as dorsal root ganglia toxicity. These approaches are characterized by mandatory immunosuppression [70,86,87]. Systemic exposure also increases the likelihood of humoral and cellular immune responses, which may reduce the efficacy and duration of transgene expression [1,46].

### 3.2. CNS-Specific Routes of Administration

In adults, the BBB prevents efficient CNS transduction upon systemic AAV administration, necessitating delivery of the vector into the cerebrospinal space or brain parenchyma.

The main clinically used routes—intrathecal, intracerebroventricular, intracisternal, and intraparenchymal administration—provide local exposure of neurons at significantly reduced doses and minimal systemic burden, lowering the risk of hepatotoxicity and complement activation while retaining the risk of local inflammation [11,28,65]. The greatest clinical experience has been accumulated for intrathecal administration of AAVrh10 and AAV9 in neurodegenerative diseases, where this route ensures more uniform transduction of the spinal cord compared to systemic delivery [53,71,88,89].

### 3.3. Local (Regional) Administration

Local routes (subretinal, intra-articular, intramuscular, intrapulmonary, intrahepatic, etc.) involve administering the vector directly into an anatomically confined zone isolated from the systemic circulation. This strategy ensures maximum vector concentration in the target tissue with minimal or completely absent systemic exposure [90,91].

Physical isolation of the vector from the circulating immune system allows avoidance of both humoral and cellular immune responses to the capsid, making such approaches the safest in terms of systemic toxicity [92]. The main risks are local, predominantly surgical in nature (mechanical tissue damage, inflammation at the injection site) and are not associated with dose-dependent burden on the liver, kidneys, or immune system. This paradigm of “immunologically isolated delivery” has found its most striking confirmation in ophthalmologic gene therapy and remains the gold standard for diseases localized to specific structures [91,93,94].

Interestingly, intravitreal administration has been associated with a lower risk of developing severe adverse events compared to subretinal administration, which can be explained by the invasiveness of subretinal injections [70].

## 4. Risk Factors and Predictors of Toxicity

The safety of AAV therapy is determined by a complex of interrelated factors, including the patient’s immunological status, molecular design of the vector, dose, age, and concomitant conditions. Their combined influence shapes an individual risk profile that must be assessed prior to therapy administration.

The primary clinical risks associated with the administration of AAV vectors in gene therapy include: fatal outcomes due to acute liver failure, cytokine storm, and cardiogenic shock; immune-mediated complications (endothelial damage, myocarditis, myositis, and TMA); as well as transaminase elevations and the presence of pre-existing neutralizing antibodies, which diminish the efficiency of transduction and therapeutic transgene expression while amplifying immune responses [95,96].

The presence of pre-existing NAbs to the AAV capsid in serum is an absolute contraindication for therapy, as even low titers completely block transduction. Seroprevalence varies depending on the serotype and region: the highest level is observed against AAV1 (~90%), and the lowest against AAV5 (~30–60%), which explains its preference in systemic therapies for hemophilia [97,98].

Vector dose demonstrates a nonlinear correlation with toxicity. Threshold values associated with complications include: >1 × 10^14^ vp/kg for hepatotoxicity and TMA with complement activation [99], and >2 × 10^14^ vp/kg for acute liver failure and neurotoxicity of dorsal root ganglia [26]. Critically, dosing based on actual body weight in patients with obesity may lead to excessive hepatic burden, justifying the transition to dose calculation based on target tissue mass [27,28].

Serotype selection directly determines the profile of organ burden: AAV9 and AAVrh10 efficiently transduce dorsal root ganglia, increasing the risk of sensory neuropathy [68,100,101]; AAV8 and AAV5 exhibit high hepatotropism, increasing the likelihood of hepatitis [102,103]; AAVrh74, despite low hepatotropism, demonstrates pronounced cardiotropism, which is associated with the risk of myocarditis at high doses [23,102].

The molecular structure of the transgene cassette also influences safety. The use of strong universal promoters enhances expression but increases the immunogenicity of the transgenic protein. In contrast, tissue-specific promoters reduce the risk of immune responses [104,105]. Furthermore, truncated protein forms, such as B-domain deleted (BDD)-FVIII or microdystrophin, although functional, may contain novel T-cell epitopes recognized by the immune system as foreign [106,107].

The baseline liver condition is a key risk modulator. Patients with pre-existing steatosis, fibrosis, or viral hepatitis have a higher likelihood of off-target uptake and immune activation following infusion, necessitating mandatory preliminary assessment of liver function. Moreover, liver diseases may impair vector uptake by cells [108,109,110,111].

Genetic predisposition influences the intensity of T-cell responses through human leukocyte antigen [107], Toll-like receptor (TLR)-9 [112], and complement components [113,114]. Although routine genetic screening has not yet been implemented, it is considered a promising tool for personalization and represents a logical direction for future research [46].

Patient age determines both efficacy and safety. In mouse models, the immature BBB and immune system allow efficient neuronal transduction, particularly of motor neurons, with minimal risk of capsid-specific T-cell responses [73,92]. In adult mice, in contrast, the mature BBB restricts delivery and primarily targets astrocytes, which constitute a structural component of the BBB [73], while immunological maturity increases the risk of immune responses and ganglia damage [115].

Finally, concomitant therapy can modulate risk. Immunosuppressants reduce hepatotoxicity but increase inflammatory infiltrates in the liver and do not always prevent antibody production [116]. Hepatotoxic drugs exacerbate liver damage, so their use should be avoided [117], while complement inhibitors require prophylactic vaccination and/or a prophylactic course of antibiotics due to the increased risk of infections [1,118].

## 5. Cases of Severe Toxicity and Fatal Outcomes of AAV Gene Therapy in Humans

As the clinical application of AAV has expanded, reports of fatal outcomes have emerged, including cases of TMA, hepatotoxicity, and severe immune-mediated reactions, raising serious safety concerns regarding the method [102,119]. Fatal outcomes have been observed in both children and adults (Table 2), often in association with high viral doses or pre-existing vulnerabilities, such as liver disease or complement system disorders [120]. Although the total number of fatal outcomes remains low compared to the number of treated patients, their unpredictable nature and severity underscore the need for careful risk assessment [121].

## 6. Strategies for Reducing AAV Therapy Toxicity

### 6.1. Prophylactic Immunosuppression Regimens

Initial AAV gene therapy trials used a reactive approach to corticosteroid administration in cases of elevated liver enzymes, indicating liver damage, which in some instances was attributed to AAV capsid-specific cytotoxic T-cell responses. Corticosteroid treatment typically resolved the transaminase elevations (ALT (alanine aminotransferase) and AST (aspartate aminotransferase)) [125,126]. Consequently, subsequent clinical trials incorporated prophylactic immunosuppression regimens involving single or a combination of pharmacotherapies. Corticosteroids bind to glucocorticoid receptors, modulating transcriptional signaling to exert broad anti-inflammatory and immunosuppressive effects. These effects are mediated through several mechanisms, including downregulating TLR expression, suppressing pro-inflammatory cytokines, and upregulating anti-inflammatory cytokines [127].

Other immunosuppressants used in AAV gene therapy include rapamycin, mycophenolate mofetil, calcineurin inhibitors, and rituximab. Rapamycin blocks the mechanistic target of rapamycin (mTOR) protein, which inhibits the proliferation of cytotoxic T-cells and the maturation of T-helper cells. At high doses, the drug also interferes with the proliferation and differentiation of B-cells [86,128]. Antimetabolites such as azathioprine and mycophenolate mofetil inhibit inosine monophosphate dehydrogenase (IMPDH), a rate-limiting enzyme in guanosine nucleotide synthesis that is upregulated in activated lymphocytes, thereby suppressing T- and B-cell proliferation [129].

Cyclosporine and tacrolimus inhibit the signaling phosphatase calcineurin, leading to the suppression of interleukin (IL)-2 transcription, which is essential for T-cell proliferation, regulatory T-cell maturation, as well as T-cell expansion and cytotoxicity [130]. The monoclonal antibody rituximab limits antibody production by targeting CD20 on B-cells to induce apoptosis [131]. Another pharmacotherapy under preclinical trials is hydroxychloroquine, which inhibits TLR9 ligand binding and subsequent signaling to prevent TLR-mediated T-cell activation and pro-inflammatory cytokines [132].

In some cases, a combination prophylactic immunomodulation regimen proves more effective. In a study two patient groups were compared following intravenous administration of a therapeutic dose of AAV9. To prevent capsid antibody formation, Group 1 (*n* = 23) received corticosteroids alone, while Group 2 (*n* = 15) was treated with rituximab plus sirolimus alongside corticosteroids (at a lower dose/shorter duration; subgroup 2B, *n* = 3, did not receive corticosteroids). As a result, Group 2 showed no significant increase in IgM or IgG antibodies against AAV9, exhibited minimal complement activation, and did not develop TMA, whereas Group 1 experienced a robust humoral response and complement-mediated TMA [133].

The safety profile of immunosuppressants must be considered to ensure that mitigation strategies do not introduce additional adverse events. Dose, schedule, and treatment duration also impact the overall safety profile. Additionally, immunosuppressed patients are more susceptible to bacterial, fungal, and viral infections, necessitating careful monitoring and a strategy for preventing or managing infectious events during immunosuppressive therapy [134]. Table 3 provides an analysis of the main immunosuppressive drugs used in gene therapy.

### 6.2. Genetic Engineering of Vector

When developing an optimal AAV therapy to minimize adverse immune reactions, several important factors must be considered. CpG islands in the vector DNA can trigger an immune response via TLR9 activation. It has been demonstrated that genomes depleted of CpG sequences can evade the TLR9-mediated adaptive immune response in mice, representing a strategy to reduce AAV-associated immunity [142]. This approach was used to generate CpG-free ITR, which, when tested in mice, resulted in a therapeutic vector with microdystrophin. The study authors suggest the vector is less immunogenic, but further research is needed to confirm immunological advantages [143]. Double-stranded RNA (dsRNA) formed due to promoter activity in ITR domains can activate TLR3 [144]. Vector modification to attenuate or eliminate the ITR promoter function could reduce dsRNA formation and diminish the immune response triggered by TLR3 activation [145].

In addition, the genetic material carried by the vector, the immune system can recognize the transgene product as foreign. Promoters can be designed to mimic endogenous expression levels of the transgene product, such that a weak promoter may provide sufficient transgene expression for efficacy while reducing toxic or immunological effects [107]. Tissue-specific promoters can be used to drive transgene expression in target cells or organs and to restrict expression in off-target tissues that could trigger an immune response [146]. Restricting foreign transgene expression to hepatocytes using a pseudotyped AAV8 vector combined with a hepatocyte-specific promoter has been shown to significantly reduce the immune response [147]. However, CD8^+^ T-cell responses against the transgene-encoded product can occur even in the absence of viral transduction and protein expression in antigen-presenting cells (APC); transgene-derived epitopes acquired by APCs from other transduced cell types can be cross-presented to prime a cytotoxic T-lymphocyte response against the transgene product [148,149].

Neutralizing antibodies against the AAV capsid can prevent binding to target cells and potentially inhibit transduction, rendering gene therapy ineffective. Modifying the AAV capsid to eliminate Nab epitopes is a strategy that can be used to enhance transduction efficiency and reduce NAb-mediated immune responses [150]. A strategy combining N-linked glycosylation changes and a mutation in the conserved phospholipase A2 (PLA_2_)-like motif has been described to generate AAV capsids. This combined approach produced AAV8- and AAVS3-derived variants with higher transduction efficiency and lower NAb sensitivity in vitro and in vivo, with in vivo antibody profiles differing from parental AAV capsids [151].

The formation of antigen-antibody aggregates can also trigger the classical complement pathway, leading to a type III hypersensitivity reaction. Capsid engineering can also be used to alter AAV tropism, reduce the virus titer required for efficient transduction, and decrease potential side effects caused by high-dose therapy [150].

Beyond natural serotypes, significant progress has been made in developing modified AAV capsids with enhanced properties for specific clinical applications. AAV11 demonstrates efficient retrograde transport from axonal terminals to neuronal cell bodies, making it particularly valuable for mapping neural circuits and targeting projection neurons in models of neurodegenerative diseases [PMID: 37365155]. AAV13-7m8 exhibits improved efficiency of localized transduction with reduced spread to off-target tissues, which is especially advantageous for precise interventions in the central nervous system, where accurate spatial control is required [PMID: 38894521]. The 32-PLUS variant was specifically engineered to efficiently cross the blood-brain barrier while demonstrating lower hepatotoxicity compared to parental serotypes, addressing a critical safety concern for systemic administration of adeno-associated virus in central nervous system disorders [PMID: 39798705]. These engineered variants represent a new generation of AAV-based vectors that combine optimized tropism, enhanced transduction efficiency, and improved safety profiles. Their development underscores the potential of rational capsid design approaches to overcome limitations inherent to natural serotypes and expand the therapeutic window of AAV-based gene therapy.

### 6.3. Re-Administration and Neutralizing Antibodies Control

Targeting immune-privileged organs may represent a potential strategy for successful re-administration of gene therapy. For instance, the CNS provides an opportunity to bypass neutralizing antibody recognition via administration into the cerebrospinal fluid or intraparenchymal injection of AAV [152]. Overcoming the effect of pre-existing immunity to AAV9 can be achieved by intrathecal administration of an alternative serotype, AAVrh.10, as demonstrated by increased transgene expression compared to re-administration of AAV9 [88]. Direct administration of AAV into the CNS can elicit a peripheral humoral immune response, but T-cell responses are mainly transient, and innate immune adverse reactions have not been associated with direct CNS administration [153,154]. Nevertheless, despite the potential for dose titration, there is limited published data on the re-administration of gene therapy in the CNS.

The lungs are considered a potential target organ for AAV re-administration due to their unique immune environment promoting tolerance to harmless particles [155]. Preclinical studies show conflicting results: in neonatal ferrets, immunosuppression enabled overcoming neutralizing antibodies to achieve successful repeat intratracheal administration [156]. In adult ferrets, repeat AAV instillation after five months also showed efficacy comparable to a single dose, despite the presence of lung antibodies; however, it was accompanied by accelerated clearance of transduced cells, indicating inflammatory complications risks [157]. Similar findings were obtained in non-human primates: serial bronchoscopic administration to immunocompetent rhesus macaques did not elicit transduction, nor did it impair transgene efficacy [158].

Subretinal AAV re-administration to the contralateral eye is a standard clinical practice, necessitated by ethical considerations and the impracticality of simultaneous bilateral injection. The interval between administrations can range from several days to many years [159]. Staggered dosing and reduced immunogenicity risk are facilitated by the ocular immune privilege, maintained by the blood-ocular barrier. However, the barrier disruption in certain pathologies allows for the study of immune responses following re-administration [160,161].

The primary barrier to AAV re-administration is immune recognition of the viral capsid, which has prompted the development of various evasion strategies [162]. One approach involves chemical modification of the capsid surface using polyethylene glycol or polymeric conjugates, such as polysaccharides and poly(N-(2-hydroxypropyl) methacrylamide), to shield vectors from neutralizing antibodies [163,164]. An alternative solution is the encapsulation of AAV within extracellular vesicles, which significantly enhances antibody resistance without reducing transduction efficiency [165]. Nevertheless, these methods are limited by product heterogeneity and quality control complexities during clinical manufacturing. Another approach involves using empty “decoy” capsids with reduced cellular uptake to sequester pre-existing antibodies prior to therapeutic vector administration [166]. Additionally, the capsid serotype switching strategy—delivering the same transgene within different serotypes—enables the bypass of antibodies elicited by the initial dose [167,168]. Despite demonstrated efficacy in preclinical studies, this approach also has several limitations hindering its widespread clinical application [162].

Temporary removal of anti-AAV antibodies in seropositive patients using immunoadsorption or plasmapheresis is one approach that may allow retreatment. The concept has been demonstrated in rodents and non-human primates, where immunoadsorption or plasmapheresis sufficiently reduced antibody levels to allow successful transduction of seropositive animals. Plasmapheresis has also been shown to reduce NAb titers to low levels in seropositive patients [169,170,171]. Although both methods remove immunoglobulins (Ig) and antibodies, immunoadsorption is more selective for specific IgGs and immune complexes, whereas plasmapheresis removes a larger volume of plasma along with its components [172].

An alternative approach is based on the use of the bacterial enzyme IdeS, which cleaves all human IgG subclasses into Fab and Fc fragments [173]. Preclinical studies in mice and primates confirmed the ability of IdeS to reduce neutralizing antibody levels, while in vitro incubation of plasma from healthy donors and Crigler-Najjar syndrome patients with IdeS resulted in reduced anti-AAV8 IgG titers in all participants [174]. However, a significant proportion of the population has pre-formed IgG antibodies against IdeS due to previous S. pyogenes infections. These IdeS antibodies may increase the risk of hypersensitivity/infusion reactions against the IdeS drug [26177518, 14976595]. Nevertheless, there are natural and synthetic homologues of IdeS that minimize the risk of immunogenicity. These are described in more detail in Section 7. In particular, Phase 1 clinical trials demonstrated that enzyme KJ103 reduced AAV2 NAbs by approximately 90% unbiased of pre-dose NAb titers [175].

### 6.4. Preclinical Approaches to Enhance Safety

One approach to mitigating model selection risks involves utilizing specialized models tailored to diverse conditions, such as genetically modified or humanized models that closely recapitulate human physiology. These models have evolved significantly and are widely used in research. Furthermore, a single animal model may prove insufficient for meeting research objectives, necessitating the use of multiple models to ensure robust and reliable outcomes. Despite the multifaceted considerations regarding animal models, they are not a panacea for generalizing results or formulating biomedical predictions. It is essential to recognize that although alternatives have advanced considerably, animal models remain the only practical choice for numerous experiments relevant to human studies [176,177].

### 6.5. Dose Reduction Technologies

Expression of the gene or protein of interest can be upregulated or downregulated to optimize and fine-tune the desired effect. A system utilizing the immunosuppressive drug rapamycin to activate responsive transcription factors has been developed [178]. In the absence of rapamycin, only minimal transgene expression was observed, indicating the potential for a highly specific and safe regulatory system.

Riboswitches- non-coding RNA molecules capable of binding specific metabolites and controlling gene expression- are also employed [179]. Compared to other regulatory systems, riboswitches offer several advantages, including compatibility with FDA-approved drugs, small RNA size, and low-to-zero immunogenicity. Notably, their minimal sequence length is particularly advantageous given the limited packaging capacity of rAAV, making them a powerful regulatory tool for gene therapy [180,181]. The versatility of RNA regulators is further demonstrated by constructs based on RNA aptamers with a cleavable poly-A signal embedded into the 5′-untranslated region of the transgene. In the absence of the small molecule, signal cleavage leads to mRNA degradation and suppression of expression, whereas its addition preserves mRNA integrity to activate the transgene expression. Combining this mechanism with alternative splicing provides more precise expression control [182].

### 6.6. AAV Premedication and Post-Infusion Patient Monitoring

To date, there are no standardized recommendations for pre-therapeutic screening for AAV application; however, a comprehensive assessment of liver status is necessary to minimize the risks of hepatotoxicity and oncogenesis. Baseline laboratory evaluation should include bilirubin, transaminases (AST/ALT), alkaline phosphatase, and gamma-glutamyl transferase (GGT), as well as prothrombin time and albumin. History taking should focus on identifying non-alcoholic fatty liver disease, viral hepatitis B and C, alcoholic and autoimmune liver pathology, which may necessitate additional testing for hepatitis. A complete blood count with differential, comprehensive metabolic panel, urinalysis, troponin-I, and creatine kinase measurement are also recommended. The frequency and duration of monitoring are determined individually, considering the severity of infusion reactions, vector dose, indications, and patient characteristics. Optimal study intervals are considered to be immediately post-administration, as well as at 1, 2, 3, and 12 months [183].

For Zolgensma, baseline screening involves determining anti-AAV9 antibody titers via ELISA (enzyme-linked immunosorbent assay) with a threshold value of ≤1:50. A key aspect of post-infusion monitoring is assessing hepatobiliary function due to the risk of acute liver injury and hepatic failure, as indicated by elevated transaminases. Platelet counts must be monitored in the first two weeks after infusion due to the risk of transient thrombocytopenia, as well as observation for the development of thrombotic microangiopathy, infusion reactions, and elevated troponin levels [184].

In subretinal Luxturna administration, the most frequent adverse reactions (incidence ≥ 5%) are ophthalmologic, including conjunctival hyperemia, cataract, increased intraocular pressure, retinal tear, dellen (corneal stroma thinning), macular hole, subretinal deposits, eye pain, and maculopathy. Immune responses and extraocular effects are minimal. Immune responses and extraocular effects are minimal; the interval between contralateral eye injections can range from 7 days to several years, with no clinically significant T-cell responses against AAV2 or *RPE65* observed. Systemic corticosteroids are administered before and after each injection to mitigate potential immune reactions [185].

Following Hemgenix infusion, most patients experience asymptomatic transaminase elevation, predominantly within the first 4 months, with potential late-onset episodes occurring up to 24 months post-infusion. All subjects develop neutralizing antibodies to AAV5, with high pre-existing titers (≥1:3212) associated with a lack of FIX expression. The most common adverse reactions (incidence ≥ 5%) are ALT increase, headache, creatine kinase increase, flu-like symptoms, fatigue, and AST elevation [186].

Roctavian administration necessitates extensive liver function monitoring, including weekly checks for 26 weeks, then every 1–2 weeks to 52 weeks, and subsequent monitoring every 3–6 months. Assessment of ALT with AST and creatine kinase (CK) is recommended, alongside monitoring for signs of neuroinflammation. FVIII activity elevation above the normal range is observed in 28% of patients. Common adverse reactions (incidence ≥ 5%) include nausea, fatigue, headache, infusion reactions, vomiting, and abdominal pain. Frequent laboratory abnormalities (incidence ≥10%) comprise elevations in ALT, AST, lactate dehydrogenase (LDH), CK, FVIII, GGT, and bilirubin [187].

Prior to Elevidys administration, testing for total anti-AAVrh74 antibodies is mandatory; infusion is not recommended for titers ≥ 1:400. Following treatment, all patients develop antibodies to AAVrh74, necessitating weekly monitoring of liver function, troponin I, and platelet counts during a 60-day corticosteroid course. The most common adverse reactions (incidence ≥ 5%) are vomiting, nausea, pyrexia, and thrombocytopenia [188].

Following Kebilidi administration, all patients exhibit elevated total binding (up to 1:204, 800) and neutralizing (up to 1:10,240) antibody titers, persisting for up to 48 weeks. The most common adverse reactions (incidence ≥ 15%) include dyskinesia, pyrexia, hypotension, anemia, hypersalivation, hypokalemia, hypophosphatemia, insomnia, and hypomagnesemia [189].

For Upstaza, clinical experience is lacking in patients with baseline anti-AAV2 antibody titers above 1:50. Following treatment, most patients develop anti-AAV2 antibodies, which stabilize or decrease over time without association with a worsened safety profile. The most common adverse effects (incidence ≥ 10%) include insomnia and dyskinesia; feeding difficulties, irritability, and hypersalivation occur with an incidence of up to 10% [190].

## 7. Prospective and Experimental Approaches to Immunomodulation

Advancing immunomodulation strategies is essential to expand AAV therapeutic potential, particularly for repeat administration. Among the most actively developed approaches are pharmacological blockade of T-cell co-stimulation, induction of antigen-specific tolerance, enzymatic degradation of pre-existing antibodies, and engineered modifications of the viral particles themselves.

Abatacept is a recombinant fusion protein consisting of the extracellular domain of cytotoxic T-lymphocyte-associated protein 4 (CTLA-4) fused to the Fc fragment of IgG1. It competitively blocks the interaction of CD80/CD86 on APCs with the CD28 receptor on T lymphocytes, disrupting the costimulatory signal necessary for naive T-cell activation. Suppression of CD4^+^ and CD8^+^ T-lymphocyte differentiation reduces T-dependent B-cell help and the production of NAbs against the AAV capsid [191,192,193]. In systemic administration studies, mice received 500 μg of recombinant CTLA-4-IgG intraperitoneally on days-3 and -1, prior to AAV8 injection, as well as on days 1, 3, and 6 post-injections. This regimen provides transient immunosuppression by inhibiting T-cell activation and neutralizing antibody production against AAV8. Compared to prednisone, abatacept more selectively suppresses T-cell immunity without affecting transgene expression or causing side effects, and it is compatible with other immunosuppressants [193].

An alternative method is the ImmTOR platform technology, comprising polylactic acid-based nanoparticles serving as a carrier for the immunosuppressant rapamycin [194]. Intracellular release of rapamycin mediates a complex immunomodulatory effect, including suppression of humoral and T-cell immune responses [195], enhancement of regulatory Treg differentiation [196], and reduction of pro-inflammatory cytokine IL-1β production [197]. In preclinical studies of inherited metabolic disorders, specifically methylmalonic acidemia, the combination of ImmTOR and AAV provided a more sustained reduction in pathological metabolites and increased therapeutic transgene expression compared to AAV monotherapy [195].

Pegcetacoplan is also a promising inhibitor of the alternative complement pathway. Preclinical studies demonstrated that C3 inhibition reduces AAV-induced complement activation. Pegcetacoplan treatment resulted in statistically significant improvements in most clinical and hematological parameters [198].

IdeS is a recombinant cysteine protease from *Streptococcus pyogenes* that catalyzes the cleavage of the heavy chains of all human immunoglobulin G subclasses in the hinge region [47,199]. A single infusion at a dose of 0.25 mg/kg provides functional inactivation of circulating antibodies within 2–6 h, creating a temporary “therapeutic window” for subsequent administration of the AAV vector administration [200]. IdeS is approved in the EU (2020) for the desensitization of highly sensitized kidney transplantation patients (EMEA/H/C/004849); its use in the context of AAV gene therapy remains experimental and requires clinical validation. Preclinical studies have also investigated related proteases, including IdeZ from *Streptococcus equi* subsp. *zooepidemicus* and the engineered dual-action enzyme IceMG, which cleaves both IgM and IgG. It has been shown that in mice passively immunized with human IgG without IdeZ, transgene expression in the liver decreased by 10- to 100-fold, but after IdeZ, it was completely restored to the level of naive mice for both AAV8 and AAV9. In naturally immune macaques, after IdeZ, AAV9 luciferase expression was restored, but the AAV genome copy number was restored less effectively than the expression. In the heart, after IdeZ, AAV9 transgene expression was statistically significantly restored, while the AAV9 genome copy number in cardiac tissue was not restored and remained low, as in untreated immune animals [201]. Despite demonstrated efficacy in preclinical models, these agents have not advanced to clinical trials [186]. Another homologue is IdeE (an IgG-degrading enzyme of *S. equi* ssp. *equi*), was selected from a species that typically does not infect humans. From a library of IdeS mutants, KJ103 was isolated for having the most stable physicochemical properties [202]. KJ103 rapidly degrades immunoglobulin G (IgG) in plasma, maintaining its low levels for approximately one week (doses of 0.25–0.40 mg/kg reduce IgG by more than 90%). In addition to cleaving free IgG, KJ103 degrades IgG contained within the B-cell receptor. This temporarily prevents the activation of memory B cells and their conversion to antibody-producing cells. Moreover, the enzyme reduces pre-existing neutralizing antibody (NAbs) titers to adeno-associated viruses (e.g., AAV2) by approximately 90%, independent of initial titer [175].

AAV delivery of immunomodulatory transgenes has been studied mainly in autoimmune models. Intra-articular administration of AAV encoding programmed death-ligand 1 (PD-L1) reduced T-cell infiltration and severity of rheumatoid arthritis in mice without systemic effects [90]. AAV-mediated expression of IL-10 demonstrated a protective effect in experimental autoimmune uveitis [203]. However, employing such strategies to overcome immune responses against therapeutic AAV vectors remains experimental.

Hepatic tolerance is a natural immunoregulatory mechanism where antigen delivery to hepatocytes promotes naive T-cells differentiation into Tregs influenced by sinusoidal endothelial and dendritic cells [204]. The most compelling data come from hemophilia A and B models, where AAV8 delivered the clotting factor gene to hepatocytes. This approach ensured sustained, therapeutically relevant protein expression without NAb production. The underlying mechanism involves the immune system perceiving the recombinant human protein expressed in the liver as “self,” triggering tolerance through the induction of antigen-specific Tregs that support long-term transgene expression [204,205,206].

For re-administration, overcoming established humoral immunity to AAV vectors is key to enhancing gene therapy accessibility and repeatability. The most promising strategies involve combination regimens consisting of plasmapheresis, IdeS, and serotype switching. Plasmapheresis mechanically reduces Nab titer, while the recombinant protease IdeS provides functional inactivation by cleaving IgG, thereby creating a “therapeutic window.” To sustain the effect, the capsid is replaced with a next-generation, non-cross-reactive variant [207,208,209,210].

The “empty capsid” strategy is based on the competitive binding of NAbs to high-dose empty capsids. This method saturates circulating antibodies, creating an “immunological window” for the subsequent therapeutic vector infusion [166]. Some studies point to potential issues. For instance, empty viral particles may themselves induce adverse effects related to the total viral load and can reduce overall dosing efficiency by occupying infusion volume without carrying a therapeutic payload [211].

Extracellular vesicle-associated adeno-associated virus vectors (exo-AAV) represent an innovative strategy. Encapsulating the vector within an exosome masks immunogenic capsid epitopes, reducing recognition by anti-capsid antibodies while simultaneously enhancing transduction efficiency [212]. Furthermore, even upon re-administration in the presence of established NAb titers, exo-AAV retains its transduction capacity, demonstrating 40-fold higher efficiency compared to standard AAV [213].

All strategies demonstrate high potential, especially combined with immunomodulatory drugs. The choice of a specific approach depends on the target organ, the patient’s immunity status and clinical necessity.

## 8. Conclusions

AAV vectors have established themselves as the most promising platform for genetic material delivery in clinical practice, as evidenced by the regulatory approval of multiple therapeutics (Glybera [withdrawn], Luxturna, Zolgensma, Upstaza, Hemgenix, Roctavian, Elevidys, Kebilidi, and Beqvez) [29,33,35,36,38,214]. Despite proven therapeutic efficacy in the treatment of monogenic disorders, safety concerns remain a critical limitation requiring systematic analysis and further optimization (Table 2).

Analysis of clinical data reveals a clear correlation between the efficacy of systemic delivery and dose-dependent toxicity. The principal mechanisms underlying adverse events include: capsid-directed immune responses with activation of capsid-specific CD8^+^ T-cells; hepatic burden due to the tropism of most serotypes for the liver; and complement system activation with associated risk of thrombotic microangiopathy [120,191]. The most serious complications documented in clinical trials remain hepatotoxicity, TMA, and neurotoxicity affecting dorsal root ganglia [26,27].

Key determinants of the safety profile include: the presence of pre-existing neutralizing antibodies; vector dose (threshold values > 1 × 10^14^ vp/kg are associated with increased risk); selection of serotype and route of administration; and the patient’s baseline condition (hepatic function, genetic features of the complement system, age). These factors necessitate mandatory evaluation during pre-therapeutic screening [3,26,69,70,108].

**Figure 2:**
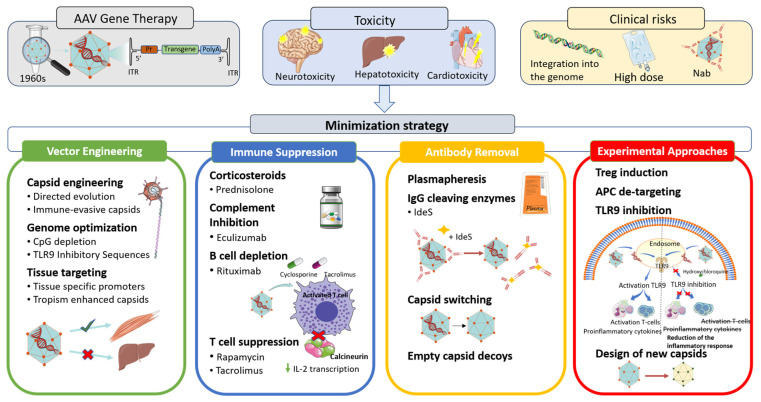
Key toxicity determinants and mitigation strategies in AAV gene therapy. Illustration from NIAID NIH BioArt Source (https://bioart.niaid.nih.gov/, accessed on 24 May 2026).

Contemporary strategies for toxicity minimization are evolving from reactive management of complications toward proactive risk mitigation (Figure 2). Standard clinical practice increasingly incorporates mandatory laboratory monitoring of hepatic function, platelet counts, and markers of myocardial injury, combined with prophylactic immunosuppressive regimens (corticosteroids, mTOR inhibitors, rituximab) [23,70,86]. In parallel, approaches based on vector engineering are advancing: design of capsids with altered tropism; CpG depletion of the vector genome to reduce TLR9 activation; and use of tissue-specific promoters to restrict transgene expression to target cells [142,146].

To address challenges associated with re-administration, methods for transient modulation of humoral immunity are under development: enzymatic degradation of antibodies (IdeS); mechanical removal of immunoglobulins (plasmapheresis); application of “decoy” empty capsids; and encapsulation of vectors within extracellular vesicles (exo-AAV). However, most of these approaches remain at the preclinical or early clinical stage and require further validation in controlled trials [166,170,173,213].

Future progress in the field depends on refinement of preclinical models for more accurate prediction of human immune responses; optimization of manufacturing processes to reduce heterogeneity of vector preparations; and development of standardized protocols for monitoring and supportive care [176,177]. Integration of improved vector constructs, rational immunosuppressive regimens, and rigorous post-infusion surveillance has the potential to expand the therapeutic window of AAV-based gene therapy, achieving an optimal balance between efficacy and safety for a broader patient population.

Moreover, the therapeutic potential of adeno-associated virus-based vectors continues to expand in the field of oncology. Recent reviews highlight the promise of using such vectors in gene therapy for melanoma, where AAV-mediated delivery of antiangiogenic factors, proapoptotic genes, immunomodulatory transgenes, or signaling pathway inhibitors may complement existing treatment approaches [215]. This diversification of AAV applications underscores the importance of further optimizing vector safety and efficacy across a broad spectrum of diseases.

## Figures and Tables

**Table 1:** Selection of AAV administration route based on disease, target tissue, and clinical limitations.

Disease	Target Tissue	The Optimal Route	Justification of the Choice	Limitations/Risks	Sources
Spinal muscular atrophy	Motor neurons of the spinal cord	Intravenous	AAV9 effectively crosses the immature BBB	High dose, hepatotoxicity, damage to dorsal root ganglia	[75,76]
Amyotrophic lateral sclerosis	Cortical and spinal neurons	Intrathecal	AAVrh10 or AAV9: Bypassing the BBB in adults, uniform transduction of the spinal cord	Risk of meningitis, limited penetration into the cortex	NCT06454682, NCT06100276
Huntington’s Disease	Striatum, bark	Intraparenchymal	AAV2: Direct delivery to the caudate nucleus and putamen of the brain	Surgical risk, limited diffusion radius	[77], NCT04120493
Hereditary retinal dystrophy	Retinal pigment epithelium	Subretinal	AAV2: High transduction, isolation from the immune system	Invasiveness, risk of retinal detachment	[78,79]
Hemophilia A/B	Hepatocytes	Intravenous	Natural hepatotropicity of AAV2/8	Risk of hepatitis, thrombotic microangiopathy and the need for immunosuppression	[80,81]
Duchenne Muscular Dystrophy	Skeletal muscles, myocardium	Intravenous	AAVrh74: The need for global muscle transduction	Very high dose, cardiotoxicity, myositis	[41,82,83]
Mucoviscidosis	Bronchial epithelium	Intrapulmonary (inhalation)	AAV2: Direct contact with the target, avoiding the liver	Mucous barrier, immune response in the lungs	[84,85]

**Table 2:** Reported cases of severe toxicity and death.

Drug/Therapy	Dose	Route of Administration	Patient Age	Complications	Time of Onset	Proposed Mechanism	Outcome	Sources
rAAV9–d*Sa*Cas9–VP64 for DMD therapy	1 × 10^14^ vp/kg	Intravenous	27 years	Mild cardiac dysfunction and pericardial effusion, followed by acute respiratory distress syndrome	After 5 days	Low patient muscle mass compared to other studies; patient received a high dose	Day 1–3 after the infusion: arrhythmia, platelet fall, B-type natriuretic peptide rise; Day 3–5 after the infusion: hypercarbia, rising troponin, precardial effusion, reduced left ventricular ejection fraction Day 6 after the infusion: acute respiratory distress syndrome and cardiac arrest Day 8 after the infusion: death	[122]
Onasemnogene abeparvovec (rAAV9) for SMA therapy	1.1 × 10^14^ vp/kg	Intravenous	4 months	Acute renal failure, hemolytic anemia, pancreatic injury, staphylococcal infection, TMA	On the 12th day	Patient had a genetic predisposition affecting complement factor I, resulting in failure to timely regulate the complement cascade	Day 8 after the infusion: thrombocytopenia (<3 × 10^9^/L), elevated lactate dehydrogenase; Day 12 after the infusion: clinical TMA (renal failure, hemolysis, schistocytes 6%) sC5b9 elevated; Day 18–25 after the infusion: response to eculizumab, normalization of sC5b9; Day 40 after the infusion: TMA markers re-increased due to sepsis (eculizumab levels are low). Death occurred following cardiac arrest, presumably due to a combination of factors including hypovolemia, sepsis, and heart failure	[119]
AAV5-GLA (AMT-191) for the treatment of the classic form of Fabry disease	Various doses: 6 × 10^13^ vp/kg, 4 × 10^13^ vp/kg, and 2 × 10^13^ vp/kg	Intravenous	Males aged 18 to 50 years (11 patients from 3 groups)	In two patients receiving the 4 × 10^13^ vp/kg dose, asymptomatic grade 3 elevation of liver enzymes was observed	Not specified	Both cases were confirmed as dose-limiting toxicities	Few weeks after the infusion: two patients on the medium-dose experienced Grade 3 liver enzyme elevations (dose-limiting toxicity); one patient on the high-dose experienced Grade 3 liver enzyme elevations (resolved). Two patients on the high dose experienced serious adverse events: chest pain, troponin elevation, and leptomeningeal enhancement. 4–12+ weeks after the infusion: Dose-dependent increase in α-Gal A persists; lyso-Gb3 is stable; 6/11 patients were withdrawn from enzyme replacement therapy. The company suspended further administration of the drug in the intermediate- and high-dose groups pending additional evaluation. Both patients responded to corticosteroid therapy and remain under follow-up	[123], NCT06270316
Resamirigene bilparvovec (rAAV8) for the treatment of X-linked myotubular myopathy	Various doses: 1.3 × 10^14^ vp/kg, 3.5 × 10^14^ vp/kg	Intravenous	Boys under 5 years of age	One of seven participants in the low-dose group died, and three of 17 participants in the high-dose group had died at the time of data collection. Causes of death in the 4 patients: hepatopathy, severe immune dysfunction and Pseudomonas sepsis, circulatory failure due to gastrointestinal bleeding and septic shock, cholestatic liver failure, liver injury	Within 1–4 weeks after initiation of the drug, all participants exhibited elevations in direct and total bilirubin above the upper limit of normal	The condition was refractory to standard immunosuppressive therapy	1–4 week after the infusion: bilirubin and liver enzyme rises; 24 weeks after the infusion: clear improvements in ventilator dependence and motor scores; 48 weeks after the infusion: some participants achieved ventilator independence; The deaths of four participants led to the suspension of the study pending investigation into the mechanism of gene therapy-associated hepatotoxicity	[124]

**Table 3:** Immunosuppressive agents in AAV gene therapy.

Agent	Molecular Target	Adverse Effects	Mode of Action	Sources
Corticosteroids	Glucocorticoid receptor	Musculoskeletal disorders, metabolic and endocrine disturbances, cardiovascular diseases	Downregulation of pro-inflammatory cytokines and chemokines	[127]
Rapamycin (sirolimus)	mTOR	Thrombocytopenia, dyslipidemia, mucositis, impaired wound healing, proteinuria	Suppression of cytotoxic T-cell and T helper cell activation, generation of regulatory T-cells (Treg), inhibition of B cell and T-cell proliferation and differentiation	[86,128,135]
Mycophenolate mofetil	Inosine monophosphate dehydrogenase type II	Gastrointestinal toxicity, leukopenia, infection, dermatitis	Inhibition of B cell and T-cell proliferation	[129,136]
Tacrolimus	Calcineurin/IL-2	Renal dysfunction, hypertension, diabetes mellitus, fever, CMV infection, tremor, hyperglycemia, leukopenia, infection, anemia, bronchitis, pericardial effusion, urinary tract infection, constipation, diarrhea, headache, abdominal pain, insomnia, paresthesia, peripheral edema, nausea, hyperkalemia, hypomagnesemia, and hyperlipidemia	Inhibition of T-cell activation and proliferation, and suppression of T helper cell-dependent B cell responses	[130,137,138]
Rituximab	CD20	Infusion-related reactions, mucocutaneous reactions, hepatitis B reactivation, progressive multifocal leukoencephalopathy, febrile neutropenia, fever, pneumonia, anemia, infection, tumor lysis syndrome	Induction of apoptosis in CD20+ B cells	[131,139]
Eculizumab	Human complement protein C5	Fever, hypertension, thrombosis, anemia	Inhibition of complement activation	[140]
Hydroxychloroquine	TLR9	Gastrointestinal disorders, retinopathy, cardiomyopathy, cardiac conduction abnormalities	Inhibition of TLR9-mediated responses to viral DNA. Inhibition of lysosomal activity, potentially preventing MHC-mediated antigen presentation	[132,141]

## Data Availability

No new data were created or analyzed in this study. Data sharing is not applicable to this article.

## Abbreviations

The following abbreviations are used in this manuscript:

AAV	Adeno-Associated Virus
ALT	Alanine Aminotransferase
APC	Antigen-Presenting Cell
AST	Aspartate Aminotransferase
BBB	Blood–Brain Barrier
BDD	B-Domain–Deleted
CK	Creatine Kinase
CMV	Cytomegalovirus
CNS	Central Nervous System
CTLA-4	Cytotoxic T-Lymphocyte Associated Protein 4
DMD	Duchenne Muscular Dystrophy
ELISA	Enzyme-linked Immunosorbent Assay
EMA	European Medicines Agency
FcRN	Neonatal Fragment Crystallizable Receptor
FDA	Food and Drug Administration (USA)
FGFR1	Fibroblast Growth Factor Receptor 1
FIX/F9	Coagulation Factor IX/its gene
FVIII	Coagulation Factor VIII
GGT	Gamma-Glutamyltransferase
HGFR	Hepatocyte Growth Factor Receptor
HSPG	Heparan Sulfate Proteoglycans
IdeS	Immunoglobulin G-Degrading Enzyme Of *Streptococcus pyogenes*
Ig	Immunoglobulin
IL	Interleukins
IMPDH	Inosine-5′-Monophosphate Dehydrogenase
ITRs	Inverted Terminal Repeats
LamR	Laminin Receptor
LDH	Lactate Dehydrogenase
LPL	Lipoprotein Lipase gene/enzyme
MHC	Major Histocompatibility Complex
mTOR	Mechanistic Target Of Rapamycin
Nab	Neutralizing Antibody
PD-L1	Programmed Death-Ligand 1
PLA_2_	Phospholipase A_2_
rAAV	Recombinant Adeno-Associated Virus
RPE65	Retinal Pigment Epithelium-Specific 65 kDa Protein
scAAV	Self-Complementary Adeno-Associated Virus
SMN	Survival Motor Neuron gene/enzyme
TLR	Toll-Like Receptor
TMA	Thrombotic Microangiopathy
Treg	Regulatory T-Cells
